# Antimicrobial-Resistant Environmental Bacteria Isolated Using a Network of Honey Bee Colonies (*Apis mellifera* L. 1758)

**DOI:** 10.1155/2023/5540574

**Published:** 2023-11-27

**Authors:** Giovanni Cilia, Ilaria Resci, Raffaele Scarpellini, Laura Zavatta, Sergio Albertazzi, Laura Bortolotti, Antonio Nanetti, Silvia Piva

**Affiliations:** ^1^Research Centre for Agriculture and Environment (CREA-AA), Council for Agricultural Research and Agricultural Economics Analysis, Via di Corticella 133, Bologna 40128, Italy; ^2^Department of Veterinary Sciences, University of Bologna, Via Tolara di Sopra, 43, Ozzano dell'Emilia (BO), 40064, Italy

## Abstract

The phenomenon of antibiotic resistance stands as a paramount health challenge in the contemporary era. Within a One Health approach, it becomes crucial to effectively track the dissemination of antibiotic resistance, not only within humans and animals but also within the environment. To investigate the environment, the honey bee (*Apis mellifera*) has emerged as a prominent environmental bioindicator due to its social, behavioral, and morphological features. The objective of this study was to describe the antimicrobial resistance (AMR) patterns of bacterial isolates from the body surface and the gut of honey bees sampled from 33 colonies throughout the Emilia-Romagna region (Italy). A total of 608 strains were examined for 19 distinct antimicrobial compounds from various classes, and the results showed that more than 50% of the isolates for eight out of nine provinces showed characteristics of nonsusceptibility toward amoxicillin and penicillin, and, generally, 98.19% of isolated strains were considered AMR and 74.67% exhibited multidrug resistance (MDR) characteristics, more frequent in Gram-negative strains (87.74%) than in Gram-positive ones (60.34%). Additionally, a significant correlation with a lower prevalence of MDR bacteria was demonstrated for one province (Ferrara, odds ratio (OR) = 3.33, (1.67; 6.64), *p*=0.0006). In conclusion, this study provides evidence for the utility of *A. mellifera* colonies as bioindicators for MDR bacteria, enabling their characterization and distribution at a geographical level. Additional investigations are required to further explore the potential role of honey bees as bioindicators for antimicrobial-resistant bacteria, particularly in terms of their association with environmental characteristics.

## 1. Introduction

Antimicrobial resistance (AMR) is a phenomenon born with antimicrobial drug discovery and use, whose exponential growth is racing with improper antimicrobial use both in human and veterinary medicine [1, 2]. The main institutions have established surveillance systems for monitoring the AMR spread in a distinct pathogen bacteria group; in Europe, it is represented by the European Antimicrobial Resistance Surveillance Network (EARS-Net) (https://www.ecdc.europa.eu/en/about-us/networks/disease-networks-and-laboratory-networks/ears-net-data, accessed June 2023). However, all these monitoring systems are limited in the sanitary contest without considering external factors, and within a One Health approach, it can be necessary to evaluate the possible spread of AMR and multidrug resistance (MDR) bacteria in the environment. To date, some studies have found AMR and antimicrobial resistance genes (ARGs) in different environments, such as rivers, embankments, and ponds, probably due to fecal contamination [3, 4]. Farming and husbandry areas can also be a potential reservoir of AMR and ARGs because soil bacteria can be influenced by animal manure derived from animals treated with antimicrobics [5–7]. Urban contexts may also be a possible source of transmission of AMR or ARGs when considering water treatment plants [8] or sanitary structures [9]. For this reason, the AMR may be regarded as an environmental factor in biomonitoring. Honey bee (*Apis mellifera* L.) colonies are commonly used as a bioindicator, thanks to the individual morphology and behavioral colony characteristics. Individually, they present a body surface covered by bristles and hairs, whereby they catch the pollen and intercept other types of particles during the flight. Concerning the behavior, they present a huge member population and an elevated number of flights of foragers per day, with a flight radius of around 1.5 km [10–12]. Considering each bee as a microsampler, the total amount of foragers bees are able to perform a representative sample of the explored environment [11]. Besides, the honey bee colonies can be managed in any type of environment, including marginal, rural, agricultural, urban, and industrialized areas [10]. All these characteristics have made honey bee colonies useful for several environmental biomonitoring plans through the analysis of bee products and/or their bodies [13–16] to detect environmental contaminants, such as heavy metals, pesticides, and microplastics [15, 17–25], environmental pollutant, such as atmospheric particulate matters [26–28], and, lately, plants, animals, human pathogens [15, 29–33], AMR and ARGs [34–39]. Honey bees can acquire AMR bacteria and ARGs during the foraging flight, especially during the ingestion of nectar and water. Besides, bacteria on flowers can be picked up by the fimbriae on their bodies during flight activity [10, 11, 27].

This study aims to describe the AMR patterns of bacterial strains isolated from the body surface and the gastrointestinal tract of honey bees, sampled from 33 colonies throughout the Emilia-Romagna region (Italy).

## 2. Materials and Methods

### 2.1. Sampling

The study was conducted as a part of the BeeNet project [40], including a network of 33 apiaries located in all provinces of the Emilia-Romagna (*Supplementary 1*). The sampling was performed in four different periods of the year, namely November 2021, March 2022, June 2022, and September 2022. All apiaries were sampled during each monthly sampling period. For each apiary, the province of origin was considered. Three hives were chosen for each apiary, and 10 returning foragers have been sampled for each hive [41, 42]. The 10 foragers have been placed in sterile 25 ml tubes type falcon and stored at 4 ± 3°C until analysis.

### 2.2. Bacterial Isolation and Identification

The bacterial isolation was made from both the body surface and the gastrointestinal tract. For bacterial isolation from the body surface, the 10 foragers belonging to the same colony were put in a 25 ml falcon tube with 6 ml of sterile physiologic solution and vortexed for 10 s [43, 44]. The consequent suspension was streaked by a 10 *μ*l sterile disposable loop onto UTIC (Condalab, Madrid, Spain) agar medium and incubated aerobically at 37 ± 1°C for 24 hr; for the gastrointestinal tract's bacterial isolation, all the 10 foragers used were dissected and for each forager, the ventriculus (small intestine and midgut) was extracted and put in 2 ml sterile microtubes with 1.5 ml of a sterile physiologic solution to create a pool with all the 10 guts. Subsequently, the pool was ground with a microbiological micro pestle and then streaked onto the UTIC medium as described above and incubated equally. After the incubation, the bacterial growth was evaluated, and the different colonies with different colors were isolated in purity onto the UTIC agar medium [45]. For the identification of isolated bacteria, each bacterial colony was subcultured in Tryptone Soy Agar (TSA) (Oxoid, Basington, UK) medium. The fresh colonies were identified using Matrix-Assisted Laser Desorption/Ionization Time-Of-Flight Mass Spectrometry (MALDI-TOF MS) (Biotyper, Bruker Inc., USA). All the colonies were identified at level species with a minimal score of 1.80 using Bruker Biotyper version 3.0 software.

### 2.3. Antimicrobial Susceptibility Testing (AST)

All the identified strains were tested for antimicrobial susceptibility to 18 antimicrobials according to the agar diffusion method described by the Clinical and Laboratory Standard Institute [46]. The 19 antimicrobials tested (see Table 1) are amoxicillin (25 *μ*g), amoxicillin/clavulanic acid (20/10 *μ*g), ampicillin (10 *μ*g), aztreonam (30 *μ*g), cefotaxime (30 *μ*g), cefoxitin (30 *μ*g), cephalothin (30 *μ*g), chloramphenicol (30 *μ*g), doxycycline (30 *μ*g), enrofloxacin (5 *μ*g), erythromycin (15 *μ*g), gentamicin (10 *μ*g), imipenem (10 *μ*g), nalidixic acid (30 *μ*g), penicillin (10 U), streptomycin (10 *μ*g), tetracycline (30 *μ*g), trimethoprim/sulfamethoxazole (1.25/23.75 *μ*g), and vancomycin (30 *μ*g). The antimicrobial susceptibility was evaluated according to clinical breakpoints provided by the European Committee on Antimicrobial Susceptibility Testing (EUCAST) or CSLI [46, 47]; the clinical breakpoints used were reported in Table 1. According to the National Reference Laboratory for Antimicrobial Resistance (Reg. 882/2004/CE) “*Istituto Zooprofilattico Sperimentale del Lazio e della Toscana* (IZS),” the intrinsic resistance of each bacterial species was evaluated and excluded from results (https://www.izslt.it/crab/wp-content/uploads/sites/8/2018/08/Tabelle-Resistenze-intrinseche-in-batteri-di-interesse-veterinario.pdf, accessed June 2023). For AST interpretation, the strains were divided into “susceptible” and “non-susceptible,” where the “non-susceptible” category included resistant and intermediate isolates. Following the definition given by Magiorakos et al. [48], isolates that were nonsusceptible to at least one antimicrobial drug were considered AMR, and isolates that were not susceptible to at least one antimicrobial drug from at least three different antimicrobial classes were considered MDR.

### 2.4. Statistical Analysis

For each isolate, data about the province of sampling (BO, RA, FC, FE, RN, MO, PC, PR, and RE) were extrapolated. Nonsusceptibility percentages for each tested antimicrobial were calculated by dividing the number of nonsusceptible strains by the number of tested strains. AMR and MDR percentages were evaluated by dividing the number of AMR/MDR strains by the number of total strains. The association between the nonsusceptibility percentages and province was tested with the Chi-squared test. The alpha risk was set to 0.05. A multivariate logistic regression was performed to assess the relation between AMR/MDR and province. Data were checked for multicollinearity with the Belsley–Kuh–Welsch technique. Heteroskedasticity and normality of residuals were assessed respectively by the White test and the Shapiro–Wilk test. A *p*-value < 0.05 was considered statistically significant. For the evaluation of the correlation between antimicrobials found to be statistically significant distributed by province and the livestock density, the Shapiro–Wilk tests were used. Pearson's coefficient was used to assess the correlation, judged very strong from 1 to 0.9, strong from 0.9 to 0.7, moderate from 0.7 to 0.5, low from 0.5 to 0.3, and poor from 0.3 to 0. The alpha risk was set to 0.05. Statistical analysis was performed with EasyMedStat (version 3.24; https://www.easymedstat.com). Bovine and swine populations were the only animal species considered, since they represent the most frequent type of livestock in the considered area (National Database of Italian Zootechnical Registry (BDN) https://www.vetinfo.it/j6_statistiche/index.html#/, accessed June 2023).

## 3. Results

### 3.1. Bacteria Isolation and Identification

**Figure 1:**
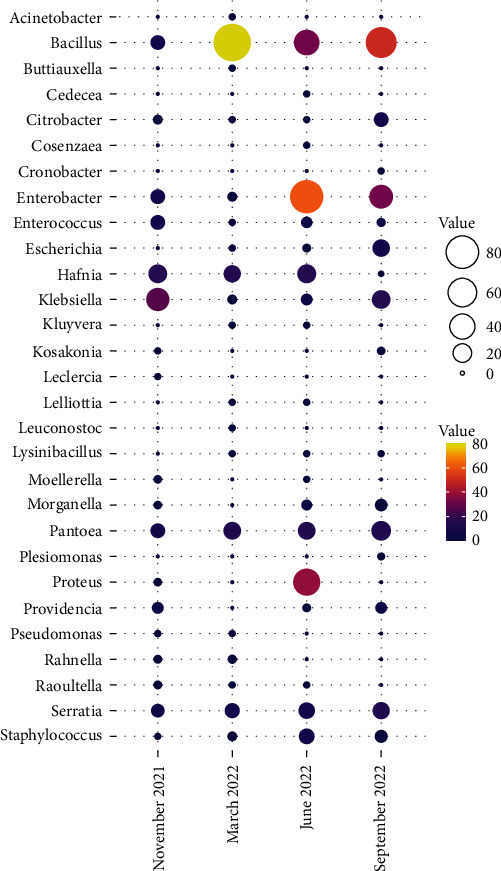
The balloon plot shows the different distributions of isolation in different sampling periods. Each dot size is representative of the relative magnitude of isolated bacteria.

A total of 608 strains, belonging to 29 genera and 79 species, were isolated. In detail, 84 strains (13.82%) were isolated from the body surfaces of foragers, while 524 (86.18%) were from the gut. Three-hundred-eight strains (52.3%) were classified as Gram-negative bacteria, while 290 (47.7%) as Gram-positive. All genera and species identified are reported in *Supplementary 2*. Considering the sampling period, a total of 97, 138, 207, and 166 bacteria were isolated, respectively, in November 2021, March 2022, June 2022, and September 2022, as shown in Figure 1.

**Figure 2:**
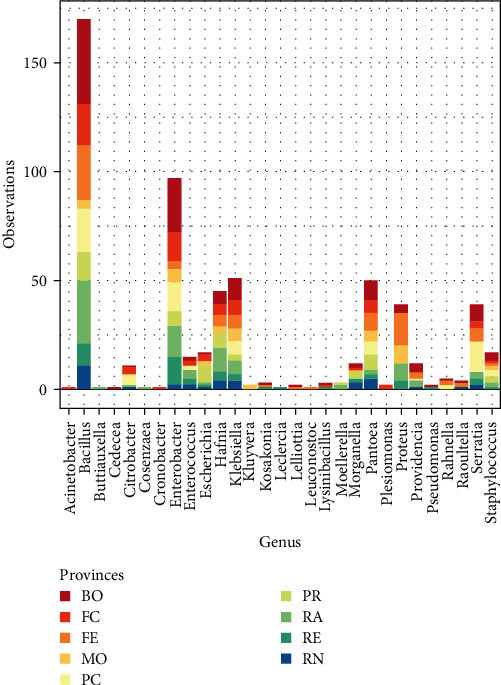
Barplot shows the number of observations for each strain belonging to each genus for each province.

The higher percentage of the strain isolated belonged to the genus *Bacillus* (27%), isolated in all provinces; the other two bacteria genera isolated in all provinces were *Klebsiella* spp., *Pantoea* spp., and *Enterobacter* spp., which represented 8.39%, 8.22%, and 15.95% of the total bacteria isolated, respectively (Figure 2).

### 3.2. Antimicrobial Susceptibility Testing and Multivariate Analysis

**Figure 3:**
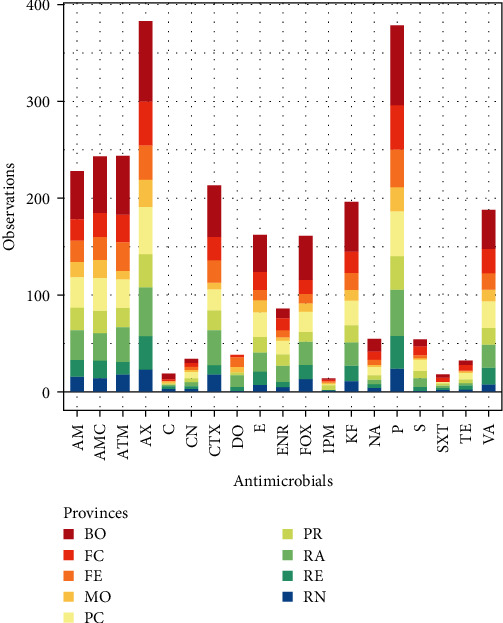
Antimicrobial resistant strains isolated per province.

AST results and percentages for each tested drug of the total number of isolated strains and other categories (Gram-positive, Gram-negative, isolation from the gut or external surface) are shown in Table 2. Five hundred ninety-seven isolates (98.19%) were considered AMR, while 454 (74.67%) were considered MDR (Figure 3).

The highest percentages of nonsusceptibility strains resulted in amoxicillin and penicillin with percentages of 62.99% and 62.34%, respectively, followed by erythromycin (59.11%), ampicillin (54.95%), and aztreonam (54.82%); these results were in line with the categories considered (Gram-positive, Gram-negative, isolation from the gut or external surface) except for Gram-positives, where the only nonsusceptibility percentages above 50% were for cefotaxime and aztreonam (Table 2).

The nonsusceptibility percentages distribution of isolates by sampling province are shown in Table 3. Among all the molecules tested, amoxicillin and penicillin resulted in higher percentages of nonsusceptibility (> 50% of strains isolated) in all the provinces but Ferrara, where the corresponding percentages were 48.75% for amoxicillin and 46.15% for penicillin. Among all provinces, the highest percentage for amoxicillin was in Modena (71.79%); instead, the highest percentage for penicillin was in Reggio Emilia (69.39%). Regarding ampicillin and aztreonam, the nonsusceptibility percentages of isolates were above 50% in six out of nine provinces (except Ferrara, Modena, and Reggio Emilia for aztreonam and Ravenna, Forlì-Cesena, and Ferrara for ampicillin). Considering erythromycin, the strains presented nonsusceptibility percentages above 50% in seven provinces except for Ferrara and Rimini, and the highest value was recorded in the Modena province, with 84.21% of isolates found to be nonsusceptible (Table 3). In the statistical analysis between the province and the single-drug nonsusceptibility, the *p*-value was considered statistically significant for FOX (*p*=0.002), AX (*p*=0.020), CTX (*p*=0.017), DO (*p* < 0.001), TE (*p*=0.003), and KF (*p*=0.010). In multivariate analysis, FE province (odds ratio (OR) = 3.33, (1.67; 6.64), *p*=0.0006) was associated with lower percentages of MDR.

**Figure 4:**
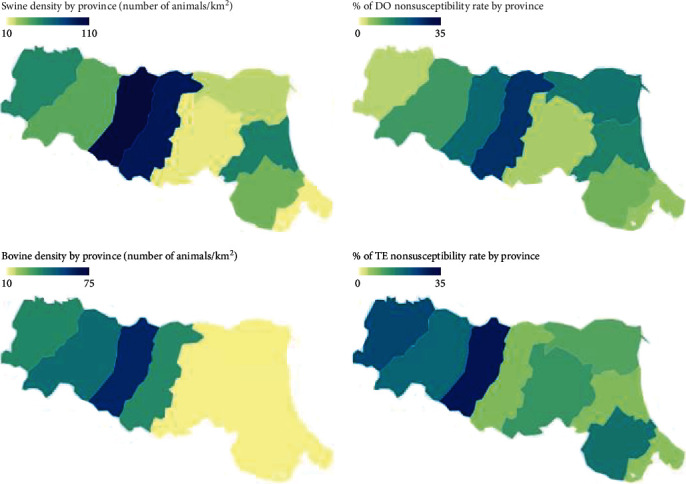
Geographical distribution of tetracyclines and TE nonsusceptibility percentages compared with swine and bovine density.

A strong positive correlation was found between TE nonsusceptibility percentages and bovine density (*ρ* = 0.74; *r*^2^ = 0.542; *p*=0.024) and between DO percentages and swine density (*ρ* = 0.72; *r*^2^ = 0.517; *p*=0.029). A strong positive correlation was found between the percentage of nonsusceptibility to at least one of the two tested tetracyclines and swine (*ρ* = 0.77; *r*^2^ = 0.591; *p*=0.016) and bovine density (*ρ* = 0.8; *r*^2^ = 0.634; *p*=0.01). These results are shown in Figure 4.

## 4. Discussion

Honey bees are widely considered excellent bioindicators due to their distinctive characteristics [13, 30, 49]. Notably, for AMR, bees have demonstrated their potential as specific indicators [34, 50], providing insights into the epidemiological distribution of ARGs.

In this study, 608 bacterial strains were isolated from bees collected from apiaries distributed across all nine Emilia-Romagna region provinces. These strains were subsequently identified; some identified species, such as *Escherichia coli*, *Klebsiella pneumoniae*, *Enterococcus faecium*, and *Staphylococcus aureus*, are widely recognized as commensal organisms but also have the potential to be pathogenic and cause infectious diseases in both animals and humans [51–55]. These species are included in the list of “priority pathogens” redacted by the World Health Organization [56], namely bacteria for which new antimicrobials are urgently needed. The possibility of comparing their susceptibility to antimicrobials between the clinical and environmental settings, using bee colonies as bioindicators, may provide insights into the indirect effects of antimicrobial use in healthcare on the environment. Furthermore, it could potentially serve as an indication for improving the management of antimicrobial treatments for specific bacterial species of interest. The results of this investigation indicated also that isolated strains genera and species change during the sampling period. According to previous studies, the bee microbiota and their colonizing bacteria strains change seasonally, probably due to different ontogenic and climate conditions, including diets and environments, which influence the growth percentages of bacteria [31, 33, 43, 44, 57, 58].

All isolated strains were tested for their susceptibility to different classes of antimicrobials, and the results revealed alarmingly high percentages of nonsusceptibility, which can be considered indicative of the regional AMR situation.

The results indicate that, in nearly all of the different antimicrobials considered, amoxicillin and penicillin exhibited higher percentages of nonsusceptibility among the isolated strains. This finding can be attributed to the fact that the penicillin class is extensively used in Italy, both in human medicine and, based on preliminary data from the Italian Drug Association, in animal husbandry as well (https://www.aifa.gov.it/documents/20142/1853258, accessed June 2023). Due to the incompleteness of these data, making an accurate comparison is challenging. Nevertheless, it still provides some insight into the use of honey bee colonies as indicators for the spread of antimicrobial-resistant bacteria to specific antimicrobials.

The resistance percentages observed against antimicrobials classified as the highest priority for human medicine by the World Health Organization are of particular concern (https://www.who.int/publications/i/item/9789241515528, accessed June 2023), such as quinolones, carbapenems, penicillins with beta-lactamase inhibitors, macrolides, and others [59]. Given that the use of antibiotics is prohibited for bees in Europe (https://eur-lex.europa.eu/legal-content/IT/ALL/?uri=celex:32004L0028, accessed June 2023), it can be excluded the possibility that the isolated bacteria have experienced selective pressure from direct drug treatments, enabling them to acquire nonsusceptible traits. Instead, these traits likely stem from surrounding environmental contamination, particularly through resources such as water and pollen. Notably, no significant differences were observed in terms of genus/species variability and AMR patterns between samples collected from the external surface and the gut, suggesting that both sites serve as valid indicators. It is worth noting that the gut provides a more favorable physiological environment for bacterial growth and colonization compared to the external surface, which was reflected in a higher number of isolates obtained from the gastrointestinal tract [49].

Generally, Gram-negative bacteria exhibited higher resistance percentages, likely attributed to their propensity for acquiring mobile resistance elements, such as plasmids, as compared to Gram-positive bacteria [60]. However, this evaluation is based on phenotypic qualitative assessment, and further genotypic analysis is necessary to confirm this hypothesis, and it should be acknowledged as a limitation of the study.

Another important aspect to consider is the variation in resistance percentages across different provinces, which underscores the potential of honey bees as bioindicators for detecting geographic differences. Although the limited number of apiaries sampled does not allow to conclude that the differences between provinces are truly representative of the real AMR situation, it can be speculated that the results from each apiary somehow reflect the area in which they are located and their sum can be considered a partial representation of the AMR situation in the province. Interestingly, one particular province (FE) exhibited statistically significant lower percentages of MDR bacteria. It is currently impossible to determine the exact origin of this specific situation, and we are unable to formulate a specific hypothesis regarding its cause. Analyzing the land use and weather conditions during the sampling period could potentially provide valuable insights and help identify explanatory variables. Indeed, previous studies have demonstrated that the urban environment, characterized by human activities, serves as a reservoir for AMR due to factors like water disposal systems and pollution [9, 61, 62]. Additionally, specific molecules, including cephalosporins (CTX, FOX, KF), tetracyclines (DO, TE), and penicillins (AX), were found to be statistically associated with provincial disparities. Once again, it is not possible to determine the exact reason for this disparity. A possibility could be to conduct a cross-analysis that might reveal a correlation between the total consumption of antimicrobials (including human and animal use) in the different provinces and the results obtained in this study. However, such data are not publicly available, and the only aspect we can emphasize is the fact that the utilization of bee colonies highlights a geographical disparity for certain antimicrobials and that these findings are important in evaluating their potential use as bioindicators. The honey bees have long been recognized as reservoirs for various tetracycline resistance genes [63, 64]. Furthermore, as shown in Figure 4 and the statistical analysis, the comparison of geographical distributions of resistance to TE and DO reveals some correlation with the density of swine and bovine farms in the respective areas (measured in animals per km^2^). This correlation has to be considered in relation to the use of this antimicrobial class in livestock. In Italy, tetracyclines are frequently used in livestock animals (they represent 23.2% of total antimicrobials sales in 2021) (https://www.salute.gov.it/imgs/C_17_pubblicazioni_3281_allegato.pdf, accessed June 2023) in both swine and bovine farms (19% and 28%, respectively). Although data about tetracyclines consumption in livestock for each province were not available, it can be speculated that the antimicrobial use is proportional to the animal density for each province and that the nonsusceptibility percentages toward tetracyclines found in bees could reflect their use in farms. Considering cephalosporins, the distribution among provinces was found to have statistical significance, but no strong correlation with bovine or swine density was identified. This lack of correlation could be attributed to the limited usage of cephalosporins in livestock, accounting for only 0.2% of total sales in 2021. As a result, it is not possible to establish a direct connection with animal husbandry. However, the influence of urban and industrial settings with high human activities can be hypothesized as a contributing factor.

In general, it can be argued that honey bees act as location-dependent bioindicators, reflecting the selective pressure exerted by antibiotics in localized areas. This phenomenon is attributed to the foraging and drinking activities of honey bees, as well as the horizontal transfer of resistance genes within their microbiota [36, 65].

## 5. Conclusions

This study indicates another use of honey bee colonies as bioindicators for human, animal, and environmental health. The observed high percentages of nonsusceptibility highlight the importance of considering this insect species within a One Health approach. Honey bee colonies can be used as a readily accessible and sensitive indicator for evaluating the geographic distribution of certain antimicrobic resistances. Specifically, the statistical association between MDR percentages and province suggests a potential use in terms of AMR location-dependent bioindicators, especially for some antimicrobials such as tetracyclines and cephalosporins, for which honey bees seemed to show a specific sensitivity. The potential link between AMR percentages in honey bees and the antimicrobial use in both humans and animals and its use for surveillance purposes still needs to be better investigated, especially in the broader context of a One Health perspective.

## Figures and Tables

**Table 1:** List of antimicrobials used, their relative classes, acronyms, concentration, and resistance breakpoint utilized.

Antimicrobials	Classes	Acronyms	Concentration	Resistance breakpoint (mm)
Amoxicillin	Penicillins	AX	25 *μ*g	≤20
Amoxicillin/clavulanic acid	Penicillin and beta-lactamase inhibitors	AMC	20/10 *μ*g	≤17
Ampicillin	Penicillins	AMP	10 *μ*g	≤16
Aztreonam	Monobactams	ATM	30 *μ*g	≤20
Cefotaxime	3rd cephalosporins generation	CTX	30 *μ*g	≤22
Cefoxitin	2nd cephalosporins generation	FOX	30 *μ*g	≤17
Cephalothin	1st cephalosporins generation	KF	30 *μ*g	≤17
Chloramphenicol	Amphenicols	C	30 *μ*g	≤17
Doxycycline	Tetracyclines	DO	30 *μ*g	≤13
Erythromycin	Macrolides	E	15 *μ*g	≤22
Gentamicin	Aminoglycosides	CN	10 *μ*g	≤14
Imipenem	Carbapenems	IPM	10 *μ*g	≤15
Nalidixic acid	Quinolones	NA	30 *μ*g	≤18
Penicillin	Penicillins	P	10 U	≤28
Streptomycin	Aminoglycosides	S	10 *μ*g	≤14
Tetracycline	Tetracyclines	TE	30 *μ*g	≤18
Trimethoprim/sulfamethoxazole	Sulphamides	SXT	1.25/23.75 *μ*g	≤15
Vancomycin	Glycopeptides	VA	30 *μ*g	≤16
Enrofloxacin	Fluoroquinolones	ENR	5 *μ*g	≤19

**Table 2:** Percentage of nonsusceptibility for each molecule tested out of the total strains isolated.

	% of Nonsusceptible	% of Gram-positive (47.7)	% of Gram-negative (52.3)	% of Ventriculum (86.2)	% of External surface (13.8)
FOX	33.61	23.27	44.44	32.27	41.43
VA	50.52	11.17	96.07	49.07	58.33
SXT	5.10	4.83	5.35	5.34	3.57
IMP	4.11	1.38	6.60	4.58	1.19
AX	62.99	37.59	86.16	63.5	59.52
AMP	54.95	31.38	86.93	54.42	57.81
AMC	52.25	27.35	79.73	51.89	54.29
CN	5.59	5.17	5.97	6.11	2.38
CTX	37.99	52.41	24.84	39.12	30.95
ATM	54.82	74.82	37.54	55.36	51.28
S	32.4	13.45	49.69	32.63	30.95
P	62.34	32.76	89.31	63.17	57.14
C	9.23	5.86	12.30	9.75	5.95
DO	11.19	13.10	9.28	11.22	10.98
TE	14.39	6.30	21.90	14.16	15.79
NA	31.93	29.20	34.28	31.71	33.33
E	59.11	31.07	91.57	58.02	65.00
KF	48.92	27.62	71.75	48.47	51.43
ENR	26.81	25.52	27.99	28.05	19.05
AMR	98.19	96.9	99.37	98.66	95.24
MDR	74.67	60.34	87.74	75.00	72.62

*Note*: Nonsusceptibility percentages considering Gram-positive and Gram-negative strains, as well as percentages considering the origin of isolation (ventricle or external surface), are reported. The percentage value of AMR and MDR for each category is given in the last two rows. In brackets, the percentage value of strains belonging to the category compared to the total strains isolated.

**Table 3:** Percentage of nonsusceptible strains in relation to the sampling provinces and percentage of AMR and MDR for each category.

	% of RA (89)	% of FC (68)	% of BO (122)	% of FE (80)	% of RN (37)	% of MO (39)	% of PC (71)	% of PR (53)	% of RE (49)	*p*-Value
FOX	34.29	30.00	46.88	14.71	43.33	25.81	38.89	22.73	41.67	0.002 ^*∗*^
CTX	41.57	36.76	48.36	30.00	48.65	17.95	38.03	37.74	28.57	0.017 ^*∗*^
KF	40.00	50.00	59.57	29.41	48.15	50.00	59.26	51.22	56.25	0.01 ^*∗*^
VA	43.86	52.08	33.61	41.86	40.00	68.42	60.42	54.84	56.25	0.388
SXT	4.49	11.76	4.92	1.25	5.41	2.56	2.82	7.55	6.12	0.239
IMP	1.12	2.94	3.28	3.75	2.70	5.13	2.82	15.09	4.08	0.055
AX	56.18	66.18	68.03	45.00	62.16	71.79	67.61	66.04	71.43	0.02 ^*∗*^
P	53.93	66.18	68.03	48.75	64.86	66.67	64.79	64.15	69.39	0.119
AMP	49.21	50.00	59.52	37.70	66.67	68.18	64.00	58.97	56.67	0.079
AMC	42.86	51.02	61.70	35.29	51.85	60.00	62.96	56.10	52.94	0.028
ATM	55.95	57.58	61.34	45.00	60.00	43.59	57.97	62.26	41.30	0.125
CN	4.49	5.88	3.28	3.75	8.11	5.13	9.86	7.55	6.12	0.686
S	25.84	39.71	31.97	17.50	27.03	33.33	39.44	41.51	42.86	0.021
C	5.62	11.94	9.84	3.75	10.81	7.69	12.68	15.09	8.16	0.398
DO	14.94	7.58	4.31	16.88	5.56	25.64	3.33	11.32	19.15	< 0.001 ^*∗*^
TE	6.49	17.19	11.93	9.68	6.06	6.67	23.33	20.00	30.23	0.003 ^*∗*^
NA	26.19	29.85	36.13	20.00	25.71	28.21	40.58	39.62	41.30	0.078
ENR	32.58	30.88	22.13	21.25	24.32	15.38	33.80	37.74	20.41	0.098
E	50.88	68.75	58.14	46.15	45.00	84.21	64.58	54.84	68.75	0.06
AMR	100.00	98.53	97.54	96.25	100.00	100.00	98.59	96.23	97.06	0.64
MDR	71.81	73.53	80.33	52.5	75.68	82.05	84.51	77.36	79.59	< 0.001 ^*∗*^

*Note*: In brackets, the number of strains isolated for each province.  ^*∗*^Statistically significant *p* value.

## Data Availability

The data used to support the findings of this study are available from the corresponding author upon request.

## Supplementary Materials

Supplementary 1 Geographical characteristics of investigated apiaries.

Supplementary 2 List of bacteria species isolated from honey bees' surface and ventriculus samples.
