# Supplementary material for: Antimicrobial-Resistant Environmental Bacteria Isolated Using a Network of Honey Bee Colonies (Apis mellifera L. 1758)
Source: Transbound Emerg Dis. 2023 Nov 27;2023:5540574. doi: 10.1155/2023/5540574 (PMC12016942; doi:10.1155/2023/5540574)
Supplement: Supplementary 1 — Geographical characteristics of investigated apiaries. [file 5540574.f1.docx]

**Table S1.** Geographical characteristics of investigated apiaries.

| **Apiary code** | **Province** | **Province Code** | **Municipality** |
| --- | --- | --- | --- |
| BOA | Bologna | BO | Valsamoggia |
| BOB | Bologna | BO | Valsamoggia |
| BOC | Bologna | BO | Sala Bolognese |
| BOD | Bologna | BO | Dozza |
| BOG | Bologna | BO | Sasso Marconi |
| BOH | Bologna | BO | Loiano |
| BOX | Bologna | BO | Bologna |
| FCB | Forlì-Cesena | FC | Cesena |
| FCC | Forlì-Cesena | FC | Forlì |
| FCD | Forlì-Cesena | FC | Bagno di Romagna |
| FCE | Forlì-Cesena | FC | Borghi |
| FCF | Forlì-Cesena | FC | Predappio |
| FEA | Ferrara | FE | Ferrara |
| FEB | Ferrara | FE | Ferrara |
| FEC | Ferrara | FE | Argenta |
| MOA | Modena | MO | Formigine |
| MOB | Modena | MO | Campogalliano |
| PCA | Piacenza | PC | Ziano Piacentino |
| PCB | Piacenza | PC | Pontenure |
| PCC | Piacenza | PC | Gragnano Trebbiense |
| PCD | Piacenza | PC | San Giorgio Piacentino |
| PRA | Parma | PR | Parma |
| PRB | Parma | PR | Montechiarugolo |
| PRC | Parma | PR | Langhirano |
| RAA | Ravenna | RA | Casola Valsenio |
| RAB | Ravenna | RA | Ravenna |
| RAC | Ravenna | RA | Ravenna |
| RAD | Ravenna | RA | Lugo |
| REA | Reggio Emilia | RE | Novellara |
| REB | Reggio Emilia | RE | Campagnola Emilia |
| REC | Reggio Emilia | RE | Viano |
| RNA | Rimini | RN | Montescudo-Monte Colombo |
| RNB | Rimini | RN | Rimini |
