# Supplementary material for: Antimicrobial-Resistant Environmental Bacteria Isolated Using a Network of Honey Bee Colonies (Apis mellifera L. 1758)
Source: Transbound Emerg Dis. 2023 Nov 27;2023:5540574. doi: 10.1155/2023/5540574 (PMC12016942; doi:10.1155/2023/5540574)
Supplement: Supplementary 2 — List of bacteria species isolated from honey bees' surface and ventriculus samples. [file 5540574.f2.docx]

**Table S2.** List of bacteria species isolated from honey bees' surface and ventriculum samples.

| **Genus** | **Species** | **Body surface** | **Ventriculum** | **Total number of isolates** |
| --- | --- | --- | --- | --- |
| *Acinetobacter* | *pseudolwoffii* |  | 1 | 1 |
| *Bacillus* | *altitudinis* | 3 | 4 | 7 |
|  | *amyloliquefaciens* |  | 2 | 2 |
|  | *badius* |  | 1 | 1 |
|  | *cereus* | 3 | 25 | 28 |
|  | *infantis* |  | 1 | 1 |
|  | *licheniformis* |  | 15 | 15 |
|  | *marisflavi* | 1 | 3 | 4 |
|  | *megaterium* | 3 | 32 | 35 |
|  | *mojavensis* |  | 3 | 3 |
|  | *mycoides* |  | 3 | 3 |
|  | *pumilis* |  | 2 | 2 |
|  | *pumilius* |  | 1 | 1 |
|  | *pumilus* | 8 | 46 | 54 |
|  | *simplex* |  | 8 | 8 |
|  | *subtilis* |  | 4 | 4 |
|  | *thuringiensis* |  | 2 | 2 |
| *Buttiauxella* | *gaviniae* |  | 1 | 1 |
| *Cedecea* | *davisae* |  | 1 | 1 |
| *Citrobacter* | *amalonaticus* |  | 1 | 1 |
|  | *braakii* |  | 3 | 3 |
|  | *freundii* |  | 5 | 5 |
|  | *koseri* |  | 2 | 2 |
| *Cosenzaea* | *myxofaciens* | 1 |  | 1 |
| *Cronobacter* | *spp.* |  | 1 | 1 |
| *Enterobacter* | *asburiae* | 1 | 7 | 8 |
|  | *bugandensis* | 1 | 11 | 12 |
|  | *cancerogenus* |  | 3 | 3 |
|  | *cloacae* | 7 | 41 | 48 |
|  | *hormaechei* | 12 | 12 | 24 |
|  | *kobei* |  | 1 | 1 |
|  | *ludwigii* |  | 1 | 1 |
| *Enterococcus* | *faecalis* | 4 | 8 | 12 |
|  | *faecium* | 1 |  | 1 |
|  | *mundtii* |  | 1 | 1 |
|  | *thailandicus* |  | 1 | 1 |
| *Escherichia* | *coli* | 1 | 16 | 17 |
| *Hafnia* | *alvei* | 5 | 40 | 45 |
| *Klebsiella* | *aerogenes* | 1 | 3 | 4 |
|  | *oxytoca* | 6 | 29 | 35 |
| *Klebsiella* | *pneumoniae* |  | 6 | 6 |
|  | *variicola* | 1 | 5 | 6 |
| *Kluyvera* | *cryocrescens* |  | 1 | 1 |
|  | *intermedia* |  | 1 | 1 |
| *Kosakonia* | *cowanii* |  | 3 | 3 |
| *Leclercia* | *adecarboxylata* |  | 1 | 1 |
| *Lelliottia* | *amnigena* |  | 2 | 2 |
| *Leuconostoc* | *mesenteroides* | 1 |  | 1 |
| *Lysinibacillus* | *fusiformis* |  | 3 | 3 |
| *Moellerella* | *wisconsensis* |  | 3 | 3 |
| *Morganella* | *morganii* |  | 12 | 12 |
| *Pantoea* | *agglomerans* | 3 | 34 | 37 |
|  | *ananatis* | 4 | 6 | 10 |
|  | *anthophila* |  | 2 | 2 |
|  | *dispersa* | 1 |  | 1 |
| *Plesiomonas* | *shigelloides* |  | 2 | 2 |
| *Proteus* | *hauseri* |  | 3 | 3 |
|  | *mirabilis* | 6 | 24 | 30 |
|  | *vulgaris* |  | 5 | 5 |
|  | *vulgaris/hauseri* |  | 1 | 1 |
| *Providencia* | *alcalifaciens* |  | 6 | 6 |
|  | *rettgeri* |  | 4 | 4 |
|  | *rustigianii* | 1 | 1 | 2 |
| *Pseudomonas* | *koreensis* |  | 2 | 2 |
| *Rahnella* | *aquatilis* |  | 5 | 5 |
| *Raoultella* | *ornithinolytica* |  | 2 | 2 |
|  | *planticola* |  | 1 | 1 |
|  | *terrigena* |  | 1 | 1 |
| *Serratia* | *fonticola* |  | 7 | 7 |
|  | *liquefaciens* |  | 2 | 2 |
|  | *marcescens* | 2 | 25 | 27 |
|  | *nematodiphila* | 1 | 2 | 3 |
| *Staphylococcus* | *aureus* |  | 1 | 1 |
|  | *capitis* | 1 | 1 | 2 |
|  | *epidermidis* | 1 | 4 | 5 |
|  | *equorum* |  | 1 | 1 |
|  | *haemolyticus* | 2 | 2 | 4 |
|  | *hominis* | 2 |  | 2 |
|  | *warneri* |  | 2 | 2 |
